# Persistence of motor-cognitive inhibition deficits in children with developmental coordination disorder (DCD): a longitudinal perspective

**DOI:** 10.1038/s41598-025-28474-9

**Published:** 2025-11-26

**Authors:** Reza Abdollahipour, Ludvík Valtr, Kamila Banátová, Lucia Bizovská, Tomáš Klein, Zdeněk Svoboda, Bert Steenbergen, Peter Henry Wilson

**Affiliations:** 1 Department of Natural Sciences in Kinanthropology, Faculty of Physical Culture, Palacký University Olomouc, Olomouc, Czech Republic; 2 Department of Pedagogical and Educational Sciences, Behavioural Science Institute, Radboud University, Nijmegen, The Netherlands; 3 Australian Catholic University, Melbourne, Australia

**Keywords:** Developmental coordination disorder, Children, Motor control, Motor inhibition, Goal-directed action, Psychology, Health occupations

## Abstract

The aim of this longitudinal study was to investigate how double-jump reaching task (DJRT) performance varies as a function of inhibitory load in children (6–12 years) with severe and moderate forms of developmental coordination disorder (s-DCD and m-DCD, respectively), compared with typically developing children (TDC). We tested 20 s-DCD, 43 m-DCD, and 192 TDC children, divided into younger (6–8 years) and older (9–12 years) age bands within each motor group. Children were tested twice, the second time after a 1-year follow-up, on two DJRT versions: standard (DJRT) and anti-jump (AJRT). Stimuli for each task appeared on a 42-inch touchscreen with a central home base and three target locations at − 20°, 0°, and 20°, 40 cm above the home base. For the DJRT, children lifted their index finger from the home base to the displayed target; 80% of trials were non-jump (to the central target) and 20% were jump where the target shifted left or right at lift-off. For the AJRT, they instead pointed to the contralateral location on anti-jump trials. Movement time difference measured the time difference between jump/anti-jump and no-jump trials. For the DJRT, there was no motor group effect, while older children made faster adjustments to jump targets. For the AJRT, the s-DCD group was consistently slower and had a significantly larger MTdiff score than both the m-DCD and TDC groups, regardless of age. The findings indicate that while cognitive-motor coupling improves with age in children with DCD, those with s-DCD have persistent difficulties.

## Introduction

Movement clumsiness in children (or Developmental Coordination Disorder—DCD) has been linked to underlying deficits in predictive motor control (aka *internal modeling deficit*)^1,2^, exacerbated by tasks that also involve a cognitive component. This underlying issue leads to a dependence on slower forms of visual and multimodal feedback, and poor motor inhibition, expressed behaviorally by slower movements with more frequent corrections compared with typically developing children. So called motor-cognitive coupling is indexed, for example, by reduced performance on anti-jump reaching tasks^3^ and dual-tasks like walking over obstacles while making a visual discrimination judgment^4^. What remains unclear is whether such deficits persist with age as a function of the severity of DCD. This paper describes a longitudinal study that compares changes in cognitive-motor coupling under different levels of inhibitory control in children with severe DCD (s-DCD), moderate DCD (m-DCD) and typically developing children (TDC).

Predictive motor control involves using forward estimates of limb position to adjust movement in real-time, especially should some perturbation occur, like a shift in a visual target or unexpected undulation in the terrain. Movement is said to be controlled by its expected consequences^5^. This is investigated using target perturbation paradigms like the double-jump reaching task (DJRT). This task traditionally examines how people adjust their movements when a target shifts unexpectedly during a reach. The target “jumps” at the point of lift-off to a new location, and the participant must quickly adjust reach trajectory online to hit the new target location. Commonly, 80% of trials target location does not shift (or jump), while 20% involve target jumps to a peripheral location (viz perturbation trials). In the case of visually-controlled action, posterior visuomotor networks of the dorsal stream (including posterior parietal cortex) are enlisted to support predictive control. At the same time, cognitive (or top-down) processes are enlisted to implement, monitor, or adjust action goals to the changing needs/motivations of the performer. For motor tasks that involve inhibition (i.e., the ability to resist movement to a prepotent stimulus like a visually-cued location), automatic motor control processes must be dampened to provide flexibility in response—a balance between auto-pilot and cognitive control. This balance is something that develops gradually over childhood, and appears to be poorly controlled in children with DCD.

Our previous studies suggest that children with DCD show particular difficulties on anti-jump reaching tasks (AJRT)—i.e., suppressing a prepotent (or dominant) response by reaching for a location contralateral to that of a cued target^1^. These children are both slower to change trajectory when implementing the anti-jump movement and take more time overall to complete anti-jump trials relative to non-jump. Interestingly, however, our longitudinal modeling between 6 and 13 years of age shows that children with DCD do tend to close the performance gap relative to TDC with maturation^1^. By 11–12 years, performance on anti-jump tasks start to mirror that seen in TDC; they better integrate internal/predictive models, sensory feedback and (top-down) inhibitory control when making online corrections, indeed, approaching adult-like performance. Notwithstanding this, their motor skills continue to unfold into adolescence.

The question of whether the difficulties we see in the online control of reaching are more pronounced in children with severe DCD remains an issue of debate. A recent study by our team showed that performance differences between TDC and DCD were more evident on the AJRT than DJRT, and occurred regardless of the DCD severity^3^. By comparison, a longitudinal study showed that children with persistent DCD (over a 2-year period) were significantly more likely to have ongoing cognitive issues^6^ than those with remitting DCD or TDC groups. In other work on eye-hand coordination, deficits in the integration of online and inhibitory control appear to be independent of DCD severity^7^; however, whether they persist with age has not been examined. Chen et al.^7^, for instance, compared moderate and severe DCD on the *covert orienting of visuospatial attention task* (COVAT: see Wilson and Maruff^8^). Precues were presented centrally (near fixation), of which 72% were valid, 18% invalid (pointed to the wrong target location), and 10% no-cue. Both DCD groups had higher invalid cue effect (ICE) values (or greater disparity in their response time between valid and invalid cues), compared with controls; as well, the difference between DCD groups approached significance. This suggested difficulties in response inhibition and attentional disengagement in children with DCD, regardless of severity. It remains unclear whether target-directed pointing differs between mild and severe DCD.

The aim of our study presented here was to enlist the double-jump reaching paradigm to investigate longitudinal changes in online (or predictive) motor control as a function of cognitive/inhibitory load and the severity of DCD. Specifically, we compared children with severe DCD (s-DCD), moderate (m-DCD), and typically developing children (TDC). We hypothesized the persistence of poor cognitive-motor coupling on the AJRT (which involves both online motor control and inhibitory components) in the younger s-DCD group, but significantly improved function in the older s-DCD group; both m-DCD and TDC groups would show improved performance in each age cohort. For the DJRT (which involves an online control component), we predicted that the s-DCD group would improve their performance with time, evident in both younger and older cohorts. Developmentally, we also expected that the performance gap between younger and older cohorts (on each task) would decline with time.

## Materials and methods

### Participants

The study included 63 primary school children aged 6–12 years with DCD, divided into 20 children with s-DCD (7 girls/13 boys, mean age = 8.43 + 1.27 years) and 43 children with m-DCD (21 girls/22 boys, mean age = 9.07 ± 1.80 years). Additionally, 192 TDC (96 girls/96 boys, mean age = 9.02 ± 1.65 years) participated, resulting in a total sample of 255 children. Each group was further divided into two age bands: younger children (6–8 years old, *N* = 124: s-DCD: 3 girls/8 boys, m-DCD: 12 girls/10 boys, TDC: 43 girls/48 boys, mean age = 7.45 ± 0.76 years) and mid-aged children (9–12 years old, *N* = 131: s-DCD: 4 girls/5 boys, m-DCD: 9 girls/12 boys, TDC: 53 girls/48 boys, mean age = 10.40 ± 0.73 years). An a priori power analysis with G*Power 3.1 indicated that 66 participants would be sufficient to achieve a power (1 − β) of 0.95, effect size ηp^2^ = 0.06 (*f* = 0.25), and an α level of 0.05^9^. Children were recruited from four primary schools in the Olomouc region and nearby areas in Moravia of the Czech Republic. In total, 255 TDC and DCD children completed both the DJRT and AJRT at two-time points with a 1-year gap. The study received approval from the Ethical Committee of the Faculty of Physical Culture, Palacký University Olomouc (FTK 46/2020), as well as from the participating schools. Prior to testing, written consent was obtained from the parents or legal guardians of the children, and verbal consent was obtained from each child.

#### Inclusion criteria

Initially, all children underwent assessment using the MABC-2 test battery. Children diagnosed with DCD met the criteria outlined in the Diagnostic and Statistical Manual of Mental Disorders, 5th edition (DSM-5-TR)^10^. The Movement Assessment Battery for Children, 2nd edition (MABC–2)^11^, recommended by the European Academy of Childhood Disability^12^, assessed their motor competence (criterion A). The adapted Czech version of the MABC–2 checklist was used by classroom teachers to evaluate persisting motor impairments in daily activities (criterion B). School psychologists reviewed each child’s medical and behavioral records to assess criteria C and D. Children who scored ≤ 16th percentile on both the MABC–2 Test and MABC–2 checklist, and met DSM criteria C and D were classified as DCD. Based on MABC-2 total scores, DCD children were divided into two subgroups: m-DCD (6th–15th percentile) and s-DCD (≤ 5th percentile). The typically developing children (TDC) group all scored above the 20th percentile in MABC-2.

#### Exclusion criteria

Children who had intellectual, physical, or sensory disabilities, or displayed symptoms of a medical condition impacting movement, were not included in the study. Twenty-one children were identified as having ADHD and were subsequently excluded. Among them, one had comorbid autism spectrum disorder (ASD), and nine had learning disorders. It is worth noting that while this screening by school psychologists helps exclude children with diagnosed conditions (e.g., ADHD, ASD), it may not identify undiagnosed children, given the high rate of co-occurring conditions in children with DCD.

### Measurement instruments

The MABC-2 was conducted in the school gym by a team of examiners who had received training and were certified experienced professionals following standardized protocols outlined in the Examiner’s Manual^11^. Standardized norms specific to the Czech population were applied for the MABC-2 Test^13^, which has demonstrated good validity and reliability across various age groups^11,14^. The MABC–2 checklist^11^ has also demonstrated excellent internal consistency (Cronbach’s α > 0.92)^15,16^, good-to-excellent inter-rater reliability (ICC = 0.78–0.91)^17^, and discriminant validity as a predictor of motor impairment^16^. The adapted Czech version of the MABC-2 Checklist is sensitive to DCD and correlates significantly with the MABC-2 Test (*r*s = − 0.31)^18^.

### Apparatus and experimental task

The DJRT was utilized to evaluate real-time motor control. This task was programmed using the VIRTOOLS Software Package^19^ and displayed on a black Iiyama 43-inch touchscreen monitor (Iiyama, Tokyo, Japan), positioned horizontally on a height-adjustable table in portrait orientation. To minimize contrast interference, all stimuli were presented against a black background. The display comprised a green “home base” circle and three yellow “target” circles, each with a diameter of 25 mm. The home base circle was centrally located at the bottom of the screen, positioned 50 mm from the bottom edge, while the three yellow target circles were situated 40 mm above the home base, spaced at − 20°, 0°, and 20° angles. The home base circle illuminated green upon contact with the index finger and deactivated upon finger lift-off. After each trial, the child returned their index finger to the home base. To prevent anticipation, target illumination was randomly delayed between 500 and 1500 ms. A successful trial was achieved when the child touched the illuminated yellow target location within its circular boundary with their index finger, resulting in the yellow light extinguishing and an auditory tone signaling trial completion. Of all trials, 80% were non-jump, with the middle yellow target circle remaining illuminated until touched by the index finger. The remaining 20% were jump trials, wherein the yellow target location shifted to the left or right peripheral location upon movement onset.

The AJRT was conducted separately but followed the same protocol as the DJRT, with the exception that children were required to touch the contralateral target location on jump trials, referred to as anti-jump trials. Twenty percent of all trials in the AJRT were anti-jump.

### Procedure

The study took place in a serene laboratory environment within the university premises, featuring standard fluorescent lighting and designed without windows to minimize any potential distractions. Hand dominance was determined by MABC-2 manual dexterity tasks and collecting self-reported information. Participants engaged in both the standard DJRT and AJRT during two separate sessions, lasting approximately 10–15 min. The DJRT session preceded the AJRT session because it was easy for younger children to learn the tasks when progressing from the simple to the more complex inhibition task^1,3,20^. In the DJRT, participants were instructed to stand behind a table adjusted to their waist height, place their index finger on the green home base circle, and swiftly reach and touch one of the peripheral yellow target circles when illuminated. During the AJRT, participants were instructed to touch the outlined circle opposite to the illuminated one, which constituted an anti-jump trial. Before each session, task demonstrations were conducted to ensure participants comprehended the objectives and actions required for non-jump, jump, and anti-jump trials. Twenty practice trials were administered in each session. Each testing session comprised 80 trials divided into two blocks of 40 trials. These included 32 non-jump trials and 8 DJRT/AJRT trials, arranged in a pseudo-random order across both left and right target locations within the 40 trials. Children completed both the DJRT and AJRT at two-time points with a 1-year gap.

### Measures and statistical analysis

For each task, we recorded the reaction time (RT) and movement time (MT) for each trial. MT was defined as the time from the lift-off—the moment the dominant hand’s index finger physically lifted from the green “home base” touch screen surface—to the moment it touched the display. A trial was deemed successful when the index finger touched within the circular boundary of the designated target, causing it to disappear and emitting an auditory signal indicating completion^1^. Trials without a response or with errors were excluded. A minimum of eight successfully completed jump/anti-jump trials per block was required^3,20^. We calculated the average MT for jump, anti-jump, and non-jump trials. In the DJRT, the assessment of online control involved comparing the movement time difference between jump and no-jump trials (MTdiff), whereas in the AJRT, the evaluation focused on the movement time difference between no-jump and anti-jump trials (AJMTdiff) to gauge the integration of online and inhibitory control^20^.

Response errors were documented for both the DJRT and AJRT tasks, comprising four types: touch-down error (TDE), indicating instances when the index finger touched areas outside the designated yellow target spot; anticipatory error (AE), occurring when the index finger was lifted from the green “home base” circle before the appearance of the yellow target or within 150 ms of the stimulus display^21^; center touch error (CTE), when the central target spot was touched instead of one of the peripheral target spots within the jump trial; and wrong-touch error (WTE), representing instances where an incorrect target spot (or cued target spot) was touched within the anti-jump trial.

For each task, normality assumptions were tested using the Shapiro–Wilk test (*p* > 0.05). Outliers were identified as scores exceeding ± 1.5 standard deviations (*SD*) from the mean^22^; specifically, if a child scored > 1.5 *SD* on either the DJRT or AJRT, their data were excluded. Furthermore, participants who failed to meet the four diagnostic criteria specified in the DSM-5, or were unable to follow task protocols due to distraction were excluded from the study. Consequently, one child with s-DCD, two with m-DCD, and five TDCs were removed from further statistical analysis. A three-way mixed between-within subject ANOVA was conducted for each task, employing a 3 (motor competence: TDC, m-DCD, s-DCD) × 2 (age groups: 6–8; 9–12 years old) × 2 (time: 1 and 2) factorial structure to compare MTdiff scores across groups. Levene’s test of equality of error variances, which assesses homogeneity of variances between groups at each time point, showed no violations of the homogeneity of variance assumption for the AJRT-MT difference scores at either season 2 or 3, nor for the DJRT MTdiff at season 3 (*p* > 0.05); the only violation was on DJRT MTdiff at season 2, *p* = 0.008. We validated the ANOVA findings for DJRT MTdiff scores at season 2 by performing an additional non-parametric Kruskal–Wallis test to examine group differences.

Error scores for DJRT and AJRT tasks including AE, TDE, WTE, and CTE were independently compared across three groups for each measurement time using the Kruskal–Wallis test. Significant differences found in error variables were further analyzed with post-hoc Mann–Whitney *U* tests between groups. The effect size for the Kruskal–Wallis test was calculated using eta squared based on the *H*-statistic: η^2^[*H*] = (*H* − *k* + 1)/(*n* − *k*), where *H* is the Kruskal–Wallis test value, *k* is the number of groups, and *n* is the total number of observations. To estimate the effect sizes in the non-parametric Mann–Whitney *U* test, the *r* effect size was calculated by dividing the obtained *z* score by the square root of the sample size number^23^. These *r* values were then transformed into the equivalent of Cohen’s *d* values using the formula *d* = [(√h)*r]/[√1 − *r*^2^] where h = [(*n*1 + *n*2 − 2)/*n*1] + [(*n*1 + *n*2 − 2)/*n*2]^24–26^. Effect sizes for ANOVA and Kruskal–Wallis tests were reported as partial eta squared (ηp^2^) or eta squared (η^2^) values, with 0.01, 0.06, and 0.14 indicating small, moderate, and large effects, respectively^25,27^. Group differences were evaluated using Cohen’s *d* and interpreted with conventional benchmarks: small (*d* = 0.2), medium (*d* = 0.5), and large (*d* = 0.8) effects^25^. Within-subject effect sizes were calculated using the repeated-measures version of Cohen’s *d*, which accounts for the correlation between time points when measuring differences between group means^28^.

A total of 48 correlation tests were conducted to examine associations between MTdiff and AJMTdiff with four error types (AE, TDE, WTE, CTE) across three groups (s-DCD, m-DCD, TDC) at two time points. To control for Type I error due to multiple comparisons, we also adjusted the significance threshold notionally to 0.01, as the Bonferroni correction is deemed too conservative^29^. Correlations between MTdiff, AJMTdiff, and AE, TDE, WTE, and CTE errors for each measurement time were estimated using Spearman’s rho correlation tests.

## Results

### Age group

A one-way ANOVA test found no significant difference in the mean age between participants in the s-DCD, m-DCD, and TDC groups, (*F*(2, 259) = 1.229, *p* = 0.294, ηp^2^ = 0.009).

#### Movement time difference

##### DJRT

**Fig. 1:**
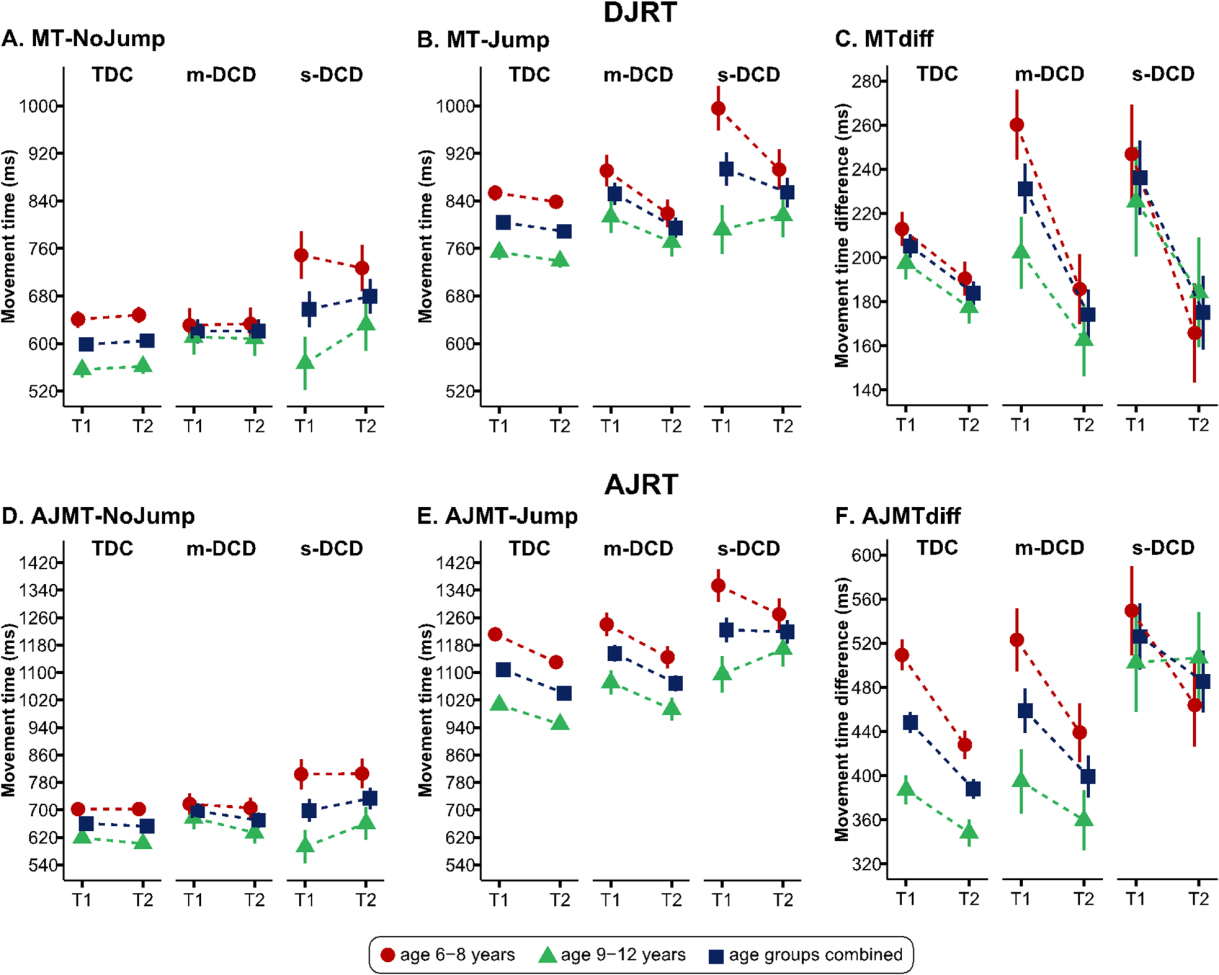
Movement time-Nojump (MT-NoJump), Movement time-Jump (MT-Jump), and movement time difference (MTdiff) for the double jump reaching task (DJRT) and anti-jump reaching task (AJRT), for each group [typically-developing children (TDC), moderate DCD (m-DCD), and severe-DCD (s-DCD), over two-time points across the age groups (younger: 6–8 years; older: 9–12 years) and age groups combined.

Mean performance on DJRT, MTdiff, MT-NoJump, and MT Jump are presented for each combination of motor group and age in Fig. 1A–C and Table 1. Regarding MTdiff, all groups had significant improvement in motor performance from time 1 compared to time 2, regardless of motor competency (*p* < 0.001, ηp^2^ = 0.125). A significant interaction between groups and time was found (*p* = 0.010, ηp^2^ = 0.036). Detailed comparisons showed significant motor performance improvements from time 1 to time 2 in the s-DCD group (*p* = 0.001, *d* = 0.485, 95% confidence interval (CI) [24.33, 97.75]), m-DCD group (*p* < 0.001, *d* = 0.569, 95% CI [32.07, 81.90]), and TDC group (*p* < 0.001, *d* = 0.291, 95% CI [9.50, 33.11]). No other significant differences were observed in the primary or interaction effects, nor in the post hoc comparisons (all *p*s > 0.05), [Kruskal–Wallis test also showed no significant difference between s-DCD, m-DCD, and TDC groups in DJRT MTdiff in season 2 (*p* = 0.140)].

**Table 1:** Analysis of the interactions of groups, measurement time, and age groups on the movement time difference in the double-jump reaching task. The group values are presented as mean ± standard error (in milliseconds).

Variable	TDC	m-DCD	s-DCD	Effect	*F*	*p*	ηp^2^
MTdiff							
T1	205.2 ± 5.40	231.1 ± 11.41	236.1 ± 16.81	G	0.51	0.601	0.004
T2	183.9 ± 5.39	174.1 ± 11.38	175.1 ± 16.77	T	35.73	< 0.001	0.125
T1, T2	194.6 ± 4.49	202.6 ± 9.48	205.6 ± 13.96	AB	2.61	0.107	0.010
T1 (AB1)	213.0 ± 7.84	260.2 ± 15.94	246.9 ± 22.55	G × T	4.71	0.010	0.036
T2 (AB1)	190.5 ± 7.82	185.7 ± 15.91	165.8 ± 22.49	AB × T	2.76	0.098	0.011
T1, T2 (AB1)	201.7 ± 6.51	223.0 ± 13.24	206.4 ± 18.73	G × AB	0.97	0.380	0.008
T1 (AB2)	197.5 ± 7.44	202.1 ± 16.32	225.3 ± 24.93	G × AB × T	1.01	0.366	0.008
T2 (AB2)	177.4 ± 7.42	162.6 ± 16.28	184.3 ± 24.87				
T1, T2 (AB2)	187.5 ± 6.18	182.3 ± 13.56	204.8 ± 20.71				

*TDC typically developing children, *m-DCD* moderate DCD, *s-DCD* severe-DCD, *MTdiff* movement time difference for double-jump, *G* group, *T* time, *AB* age band, *T1* time 1, *T2* time 2, *AB1* age 6–8 years, *AB2* age 9–12 years.

##### AJRT

Mean performance on AJMTdiff, AJMT-NoJump, and AJMT-Jump scores are presented for each combination of motor group and age in Fig. 1D–F and Table 2. Post hoc comparisons indicated a larger AJMTdiff in s-DCD as compared with m-DCD (*p* = 0.028, *d* = 0.478, 95% CI [6.01, 147.19]) and TDC (*p* = 0.002, *d* = 0.647, 95% CI [26.29, 149.00]), regardless of the measurement time. Also, children aged 9–12 years old displayed faster AJMTdiff compared to those aged 6–8 years (*p* = 0.001, *d* = 0.710, 95% CI [29.20, 108.79]).

**Table 2:** Analysis of the interactions of groups, measurement time, and age groups on the movement time difference in the anti-jump reaching task. The group values are presented as mean ± standard error (in milliseconds).

Variable	TDC	m-DCD	s-DCD	Effect	*F*	*p*	ηp^2^
AJMTdiff							
T1	448.2 ± 9.70	458.9 ± 20.47	526.1 ± 30.15	G	5.94	0.003	0.046
T2	388.0 ± 9.03	399.4 ± 19.07	485.4 ± 28.09	T	15.67	< 0.001	0.059
T1. T2	418.1 ± 7.79	429.1 ± 16.45	505.7 ± 24.23	AB	11.66	0.001	0.045
T1 (AB1)	509.3 ± 14.07	523.1 ± 28.61	549.5 ± 40.46	G × T	0.17	0.847	0.001
T2 (AB1)	427.9 ± 13.10	439.1 ± 26.65	464.0 ± 37.69	AB × T	5.03	0.026	0.020
T1. T2 (AB1)	468.6 ± 11.30	481.1 ± 22.99	506.8 ± 32.51	G × AB	1.95	0.145	0.015
T1 (AB2)	387.0 ± 13.35	394.7 ± 29.28	502.6 ± 44.72	G × AB × T	0.24	0.785	0.002
T2 (AB2)	348.2 ± 12.44	359.7 ± 27.28	506.9 ± 41.67				
T1. T2 (AB2)	367.6 ± 10.73	377.2 ± 23.53	504.7 ± 35.94				

*TDC typically developing children, *m-DCD* moderate-DCD, *s-DCD* severe-DCD, *AJMTdiff* movement time difference for anti-jump, *G* group, *T* time, *AB* age band, *T1* time 1, *T2* time 2, *AB1* age 6–8 years, *AB2* age 9–12 years.

In addition, all motor groups showed significant improvement in motor performance from time 1 compared with time 2 (*p* < 0.001, *ηp*^2^ = 0.059). As well, younger children (aged 6–8 years) improved significantly in motor performance from time 1 to time 2 (*p* < 0.001, *d* = 0.472, 95% CI [47.38, 119.98]), regardless of motor competency. Finally, older children (aged 9–12 years) had significantly lower AJMTdiff scores relative to younger children (aged 6–8 years) (*p* < 0.001, *d* = 0.883, 95% CI [49.74, 148.77]) at time 1.

No other significant main, interaction, or simple effects were evident (all *p*s > 0.05).

### Errors

**Fig. 2:**
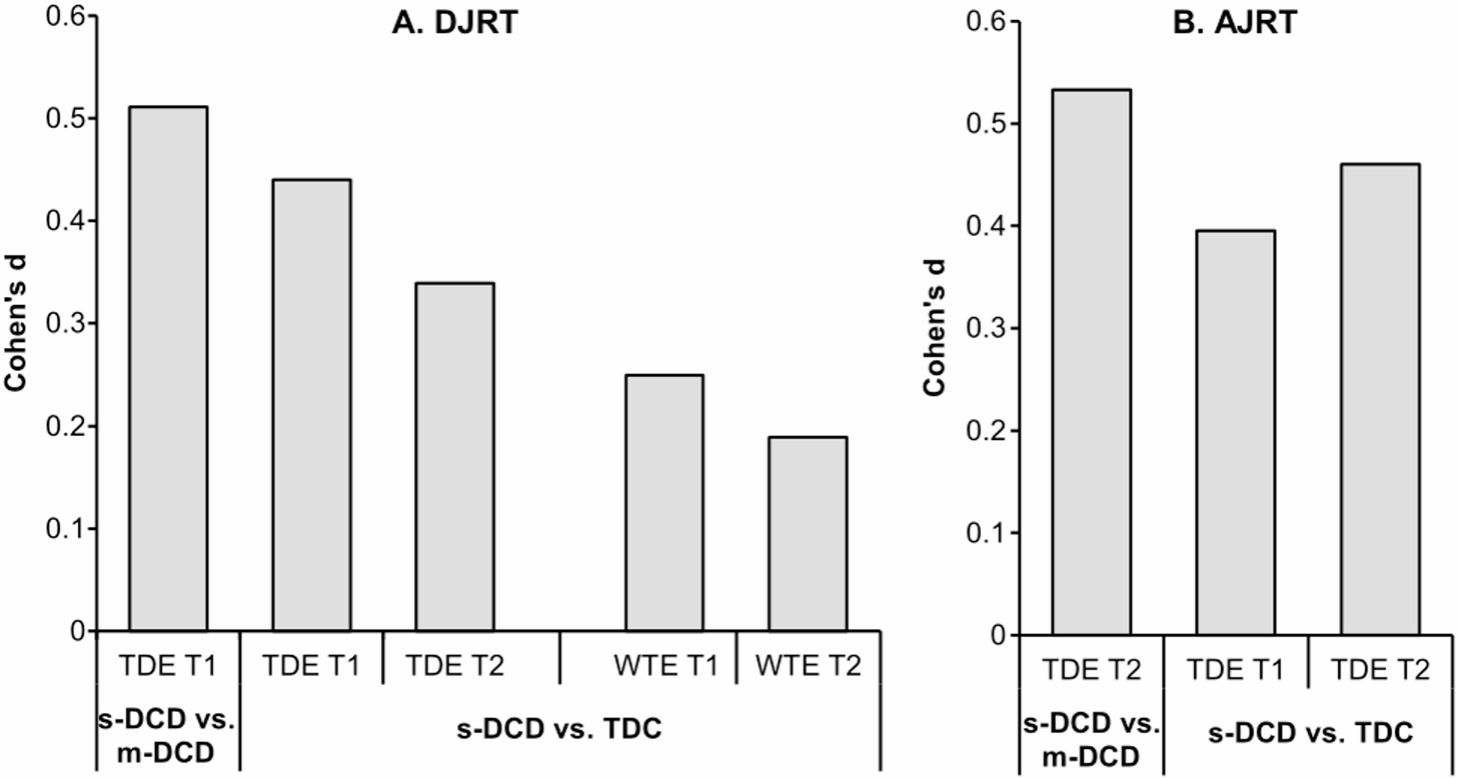
Effect size estimates, measured using Cohen’s d, for significant differences in wrong-touch error (WTE) and touch-down error (TDE) between severe DCD (s-DCD) and moderate DCD (m-DCD), as well as between s-DCD and typically-developing children (TDC) during the double-jump reaching task (DJRT) and the anti-jump reaching task (AJRT) across both measurement time 1 (T1) and time 2 (T2), regardless of age groups.

For both DJRT and AJRT, the effect sizes comparing significant differences between s-DCD, m-DCD, and TDC on errors in each time point, regardless of age groups are presented in Fig. 2.

### Correlations between MTdiff and errors across the measurement time

In DJRT, a significant correlation was found between MTdiff and CTE in m-DCD at time 1 (*r*s = 0.405, *p* = 0.007). At time 2, MTdiff correlated significantly with WTE (*r*s = 0.373, *p* = 0.014) and TDE (*r*s = 0.460, *p* = 0.002) in m-DCD, and with TDE (*rs* = *0.230,* *p* = 0.001) in TDC. After alpha-adjustments (*p* < 0.01), the correlations between MTdiff and TDE in m-DCD at time 1, m-DCD, and TDC at time 2 remained significant.

In AJRT at time 1, AJMTdiff significantly correlated with AE (*r*s = 0.201, *p* = 0.005) and WTE (*r*s = 0.142, *p* = 0.049) in TDC, and with TDE (*r*s = 0.398, *p* = 0.008) in m-DCD. At time 2, AJMTdiff showed significant correlations with AE (*r*s = 0.264, *p* < 0.001) and TDE (*r*s = 0.296, *p* < 0.001) in TDC, and with TDE in m-DCD (*r*s = 0.416, *p* = 0.005). After alpha-adjustments (p < 0.01) most correlations remained significant (*p* < 0.01), with the exception of the AJMTdiff–AE correlation in TDC at time 1 (*p* > 0.01).

## Discussion

The present study aimed to explore the persistence and development of cognitive-motor coupling in children with and without DCD, and its variation with severity of DCD and age. Specifically, we investigated whether children with s-DCD, m-DCD, and TDC exhibited differences in predictive motor control, measured via the DJRT and AJRT at two time points, 1-year apart. Our findings support the hypothesis that online motor and inhibitory control in children improves with age (over a 1-year period) on both the DJRT and AJRT, irrespective of the initial level of motor competence. Notably, children with s-DCD were generally slower to complete target-directed reaching movements than m-DCD and TDC, regardless of assessment time; this suggests that the severity of DCD may impact the development of cognitive-motor coupling, and that their challenges persist with time.

Interestingly, no performance differences were detected between s-DCD, m-DCD, and TDC groups on the DJRT—a task involving rapid, real-time adjustments based on predictive estimates of limb movement (see Abdollahipour et al.^3^); indeed, all groups showed improved performance over time, but of varying effect size. Importantly, while both s-DCD and m-DCD groups improved, the extent of improvement differed from the TDC group. These improvements correspond to the notion that rapid online control of movement tends to develop with age in children^20,30^, regardless of children’s motor competence. Indeed, previous studies^20,30^ have indicated that the ability to rapidly control movements online is influenced by age as children aged 6–7 years exhibited poorer MTdiff compared to those aged 8–9 years and 10–12 years, with no significant differences between the older groups. However, the rate at which rapid online control develops may depend on a child’s motor competence, suggesting that children with DCD may catch up more quickly in performing tasks requiring eye-limb coordination (e.g., DJRT) as compared to the tasks requiring the integration of motor and cognitive processes (e.g., AJRT).

For the AJRT, consistent performance differences over time on MTdiff scores between the s-DCD group and both m-DCD and TDC groups) indicates that severe motor difficulties may hinder cognitive-motor coupling, regardless of age. These findings align with previous studies indicating that children with s-DCD struggle to suppress automatic motor responses, particularly in the context of tasks that require high levels of cognitive inhibition^3^. Additionally, older children (ages 9–12) outperformed younger children (ages 6–8) on the AJRT (indexed by AJMTdiff), indicating developmental gains in cognitive-motor coupling, speed and efficiency as children age. All groups, regardless of age or motor competency, demonstrated significant improvements over the 1-year retest interval, with younger children (6–8 years) showing the largest gains. This age-based improvement suggests sensory feedback integration, internal modeling, and cognitive control show steady age-related change over childhood. However, the developmental trajectory in the s-DCD group was less pronounced than either m-DCD or TDC groups, indicating that difficulties with cognitive-motor coupling (i.e., inhibitory-motor, more precisely) continue over childhood for those with more severe motor impairments.

The correlational analysis confirms the moderate association between temporal and spatial accuracy metrics in m-DCD and TDC groups. In general, for both these groups, more TDEs were associated with higher difference scores (whether MTdiff or AJRTdiff). This suggests that, for both groups, spatial precision was associated with response timing, perhaps reflective of Fitts’ Law. As well, the performance gap between children with m-DCD and their typically developing peers tends to narrow over time on both spatial and temporal aspects of motor control. The absence of any associations for the s-DCD group may reflect greater variability in response strategy, specifically the prioritization of speed and/or accuracy. Children with DCD use a variety of strategies to manage motor and cognitive tasks^31^. Specifically, s-DCD children appear less concerned about making errors, prioritizing temporal speed over error avoidance, as reflected in their higher number of TDEs.

Our findings suggest that children with s-DCD (irrespective of age) are more disadvantaged by tasks involving inhibitory control, specifically the integration of motor inhibition and rapid online motor control. From a mechanistic standpoint, online visuomotor control is subserved by the posterior/dorsal stream which encompasses the posterior parietal cortex and its reciprocal connections to the motor cortex. Motor inhibition enlists the frontal executive and dorsal visuomotor systems. The integration of these systems when complex reaching (or eye-hand coordination) movements are performed under cognitive load, is not well developed in DCD (esp. s-DCD). Inhibitory control per se enlists the prefrontal and cingulate brain regions^32,33^. More specifically, the inferior frontal regions are involved mainly in global inhibition, while the superior frontal regions facilitate action restraint^34^. Taken together, developmental delays or persistent immaturity in the coupling of frontal executive and posterior visuomotor systems may explain why children with severe DCD find it challenging to update and inhibit motor actions in response to dynamic environmental changes^1^. Future research should continue to map the developmental trajectory of cognitive-motor coupling in children of varying DCD severity; this is with a view to refining our knowledge of how specific neural mechanisms contribute to the integration of predictive and inhibitory motor control, particularly in s-DCD.

### What this paper adds?

This study provides new insights into the persistence of cognitive-motor difficulties in children with s-DCD. We present one of the very few longitudinal analyses of the development of motor-cognitive coupling in children with moderate- and severe-DCD using a visually-guided pointing task. The 1-year follow-up revealed persistent performance deficits in severe s-DCD relative to m-DCD and typically-developing controls, suggesting a developmental issue with the integration of predictive motor control and response inhibition. Children with a diagnosis (or suspected) of severe DCD would benefit from assessment on a combination of motor, cognitive, and cognitive-motor integration measures of performance. This can inform more tailored intervention strategies, including cognitive scaffolding.

## Data Availability

The datasets used and/or analysed during the current study are available from the corresponding author on reasonable request.
